# Application of platelet-rich plasma in spinal surgery

**DOI:** 10.3389/fendo.2023.1138255

**Published:** 2023-03-15

**Authors:** Hengyi Wang, Jianshu Zhu, Yuanliang Xia, Yuehong Li, Changfeng Fu

**Affiliations:** Department of Spinal Surgery, The First Hospital of Jilin University, Changchun, China

**Keywords:** platelet-rich plasma, spinal diseases, cytokines, osteogenesis, facilitated repair

## Abstract

With the aging of the population and changes in lifestyle, the incidence of spine-related diseases is increasing, which has become a major global public health problem; this results in a huge economic burden on the family and society. Spinal diseases and complications can lead to loss of motor, sensory, and autonomic functions. Therefore, it is necessary to identify effective treatment strategies. Currently, the treatment of spine-related diseases includes conservative, surgical, and minimally invasive interventional therapies. However, these treatment methods have several drawbacks such as drug tolerance and dependence, adjacent spondylosis, secondary surgery, infection, nerve injury, dural rupture, nonunion, and pseudoarthrosis. Further, it is more challenging to promote the regeneration of the interstitial disc and restore its biomechanical properties. Therefore, clinicians urgently need to identify methods that can limit disease progression or cure diseases at the etiological level. Platelet-rich plasma (PRP), a platelet-rich form of plasma extracted from venous blood, is a blood-derived product. Alpha granules contain a large number of cytokines, such as platelet-derived growth factor (PDGF), vascular endothelial growth factor (VEGF), epidermal growth factor, platelet factor 4 (PF-4), insulin-like growth factor-1 (IGF-1), and transforming growth factor-β (TGF-β). These growth factors allow stem cell proliferation and angiogenesis, promote bone regeneration, improve the local microenvironment, and enhance tissue regeneration capacity and functional recovery. This review describes the application of PRP in the treatment of spine-related diseases and discusses the clinical application of PRP in spinal surgery.

## 1 Introduction

**Scheme 1:**
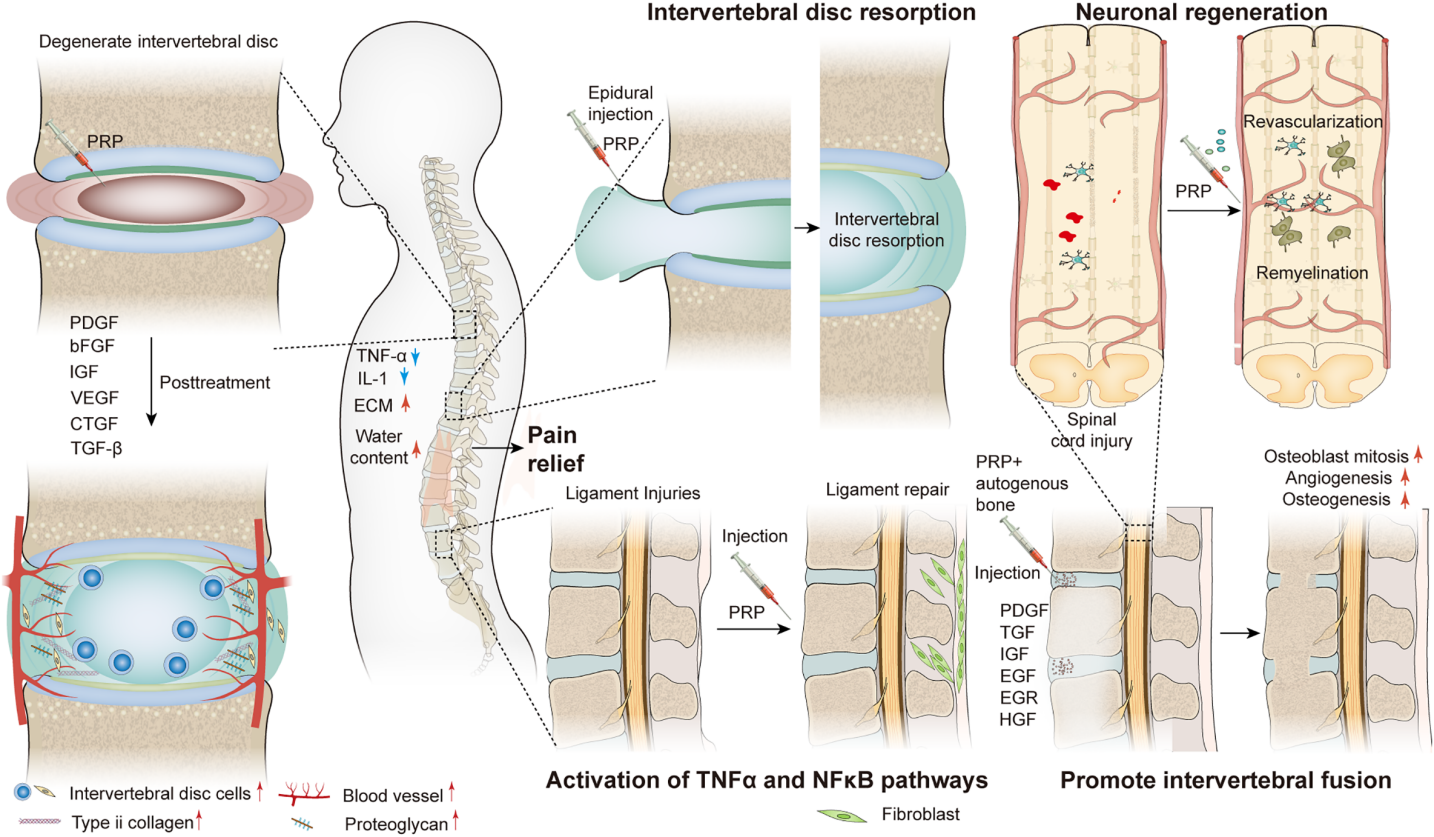
Schematic diagram of PRP in the treatment of spine-related diseases.

Spine-related diseases place a heavy financial and psychological burden on the general population (1). These diseases include discogenic low back pain (DLBP) (2), lumbar disc herniation (3), lumbar disc degeneration (4), spinal cord injury (5), ligament injury (6), and sacroiliac joint disease (7). The complexity of spine-related diseases depends on numerous factors such as age, sex, occupation, and lifestyle. Since 1990, there has been a sharp increase in the prevalence of spine-related diseases, which has become one of the main causes of disability in the world (8). At present, treatment options for these diseases can be divided into conservative, surgical, and minimally invasive interventional treatments. However, these treatment methods have several drawbacks such as adjacent segment disorder, pseudarthrosis, postoperative recurrence, secondary operation, drug dependence and tolerance, infection, dural rupture, nerve injury, and expensive treatment, among others. Moreover, the above treatment schemes are not satisfactory in promoting intervertebral disc regeneration and restoring its biomechanical properties such as damping and vibration reduction. Therefore, finding an effective and fundamental treatment plan has become a new research direction to resolve these issues, and can be beneficial for the development of spine surgery. As a natural cytokine pool (**Table 1**), platelet-rich plasma (PRP) promotes endogenous healing and may be an effective treatment for spine-related diseases (**Scheme 1**).

**Table 1:** Cytokines in platelet-rich plasma.

Cytokines	Function
PDGF **(** 9 **)**	Stimulating chemotaxis of macrophages and neutrophils; Regulate collagenase secretion and collagen synthesis; Stimulate mitosis of mesenchymal cells and osteoblasts; Stimulate chemotaxis and mitosis of fibroblasts/glia/smooth muscle cells.
VEGF **(** 10 **)**	Promote angiogenesis and increase vascular permeability; Promote endothelial cell migration and proliferation; Promote granulation tissue formation.
TGF-β **(** 11 **)**	Promote angiogenesis; Increased proliferation of fibroblasts and keratinocytes; Metalloproteinase inhibitors; Mediate extracellular matrix and collagen production; Regulating mitosis of other growth factors; Inhibit macrophage and lymphocyte proliferation.
EGF **(** 12 **)**	Increase the proliferation and migration of keratinocytes and fibroblasts; Stimulate endothelial cell mitosis; Promote granulation tissue formation.
FGF **(** 13 **)**	Promote mitosis, growth and differentiation of mesenchymal cells, chondrocytes and osteoblasts; Promote fibroblast migration; Initial stimulation of angiogenesis.
PF-4 **(** 14 **)**	Calls leucocytes and regulates their activation. Microbiocidal activities.
IGF-1 **(** 15 **)**	Increases chemotaxis of fibroblasts and stimulates protein synthesis; enhances bone formation through proliferation and differentiation of osteoblasts.

## 2 Current understanding of PRP

### 2.1 Definition and origin of PRP

As a biological product, PRP is a platelet concentrate obtained by centrifugation of autologous peripheral blood (16). PRP releases high concentrations of bioactive molecules. Numerous growth factors, cytokines, and chemokines are present in their alpha granules, which play an integral role in regulating the extracellular matrix (ECM), promoting angiogenesis and accelerating cell recruitment, proliferation, and differentiation (17). PRP has been used in maxillofacial surgery because of its positive anti-inflammatory and cell proliferation properties (18). Furthermore, with the advancement and development of technology, the application of PRP appeared in the field of sports injuries (19). Additionally, PRP has been used in the fields of urology, gynecology, orthopedics, cardiology, and ophthalmology (20–24). In recent years, in spine surgery, the application of PRP has gradually become a new therapeutic tool for the treatment of spine-related diseases.

### 2.2 Preparation of PRP

PRP can be prepared in several ways, and different PRP can be produced according to different parameters (centrifugal force, centrifugation time, and temperature) (25). At present, PRP preparation technology is generally divided into two types: one-time and two-time centrifugation. The platelet concentration and PRP activity obtained using different centrifugation times, number of centrifugation cycles, and centrifugal force are different, and the recommendations by various researchers are also different. At present, most researchers believe that PRP obtained by secondary centrifugation has a higher platelet recovery rate. Mazzucco et al. compared the two PRP preparation methods and found that platelet and growth factor concentrations were significantly higher in the two-time centrifugation method than in the one-time centrifugation method (26). Harrison et al. found that a single centrifugation method, although capable of extracting PRP, was not optimal because it resulted in decreased platelet production (27). The IADVL Dermatology Association recommends the use of the manual double-spin method for the preparation of PRP. The recommended centrifuge parameters are 100–300 ×*g* for 5–10 min for the first centrifugation and 400–700 ×*g* for the second centrifugation for 10–17 min. The PRP obtained using this method is optimal for various dermatological indications (28). Similar studies have also found that the double-spin method is more helpful for treatment of female hair loss (29). Currently, commercial equipment or kit extractions for PRP have become a trend. Different brands have developed different separation methods, and the content of the extracted PRP component is also different. Therefore, the best kit can be selected for extraction according to different therapeutic requirements (30).

### 2.3 Different types of PRP

Owing to the lack of uniform standards in the preparation methods and quality control, the prepared PRP components are different. This leads to the difficulty in comparative analysis of clinical results, and is also part of the reason for the difference in clinical efficacy. Therefore, it is urgently necessary to develop a method to unify the production standard of PRP. In 2009, Ehrenfest et al. first proposed a classification method for platelet concentrates that combines three main variables—platelet, leukocyte, and fibrin content—and divided many PRP products into four categories: pure PRP (P-PRP), leukocyte-rich PRP (L-PRP), pure platelet-rich fibrin (PRF, P-PRF), and leukocyte-rich PRF (L-PRF) (31). In 2016, Magalon et al. proposed a more comprehensive classification method, the DEPA (Dose, Efficiency, Purity, Activation) classification, which is based on four different parameters: injection dose, production efficiency, purity, and activation process. However, the DEPA classification method also has shortcomings, such as a lack of clinical correlation (32). To standardize PRP, the ISTH Science and Standardization Committee established an expert working group to reach a series of consensus recommendations and develop a new PRP classification system (33). The above classification methods aim to establish a common unified standard to achieve the standardization of PRP and lay a foundation for the further clinical application of PRP.

### 2.4 Mechanism of action and main components of platelet-rich plasma

The secretion of growth factors in PRP must be initiated by blood coagulation; therefore, anticoagulants should be used during treatment to prevent coagulation and maintain a stable state to ensure that they are not activated prior to use (34). This process inhibits clotting by disabling calcium ions, which are required in the coagulation cascade (35). This disabling step can be performed using citrate ions, which can combine with calcium ions to form calcium citrate and deactivate it (36). This is an indispensable and important condition for PRP preparation.

For downstream use, PRP must be reactivated. Exogenous calcium activators can be added to PRP to promote the release of biologically active proteins such as growth factors (37). This step can be accomplished with calcium activators such as calcium chloride, calcium gluconate, or thrombin (38). Calcium previously bound to anticoagulants can be replenished in this manner.

PRP preparation contains a variety of cytokines, growth factors, cell adhesion molecules, and chemokines. These factors participate in the process of tissue healing and proliferation, as well as the activation and synthesis of essential products through interaction and mutual regulation (39). When PRP is activated, a large number of growth factors, such as transforming growth factor-β (TGF-β), platelet-derived growth factor (PDGF), fibroblast growth factor (FGF), hepatocyte growth factor (HGF), and vascular endothelial growth factor (VEGF), can be released. PDGF can stimulate endothelial cell growth, promote capillary angiogenesis, and stimulate mononuclear macrophage chemotaxis to increase collagen synthesis. Both TGF-β and PDGF can increase the proliferation of various cells involved in wound repair, stimulate collagen synthesis, and activate the interaction between macrophages and other cytokines. VEGF is a strong vascular growth factor, which can bind to the corresponding receptors on the surface of vascular endothelial cells, stimulate the proliferation of endothelial cells, induce the formation of new blood vessels, and increase the permeability of blood vessels, especially small blood vessels. It provides nutrients for cell growth and the establishment of new capillary networks, plays an important role in wound healing and vascularization, and is a key promoter in the early stage of angiogenesis (40). In addition to growth factors, PRP also contains fibrin, fibronectin, and hypolenin, which form a fiber network and can perform the scaffold function of tissue-repairing cells, promoting cell adhesion and preventing cell loss (39). Owing to the coaction of bioactive factors in PRP, it plays a powerful role in tissue regeneration and repair.

## 3 Application of PRP in spine surgery

### 3.1 PRP for treatment of discogenic back pain

One of the most common diseases of the spine is low back pain, which can be experienced at different degrees in different stages of life. Lumbar muscle strain, intervertebral disc herniation, isthmus, spinal deformity, and lumbar spondylolisthesis are all causes of low back pain, and these factors can exist alone or in combination (41). Discogenic back pain referred to in this article is chronic low back pain caused by stimulation of the pain receptors in the intervertebral disc due to intraductal disorders, such as degeneration, intraductal fissure, and discitis, without root symptoms, nerve root compression, or excessive displacement of vertebral body segments (42). This can be described as chemically mediated discogenic pain. Increased levels of leukotriene, nitric oxide, lactic acid, and prostaglandin E in early intervertebral disc degeneration (IVD) are considered strong chemical injurious stimuli (43). The mechanism of pain is caused by the growth of vascularized granulation tissue and pain nerve fibers into annulus fibrosus or even nucleus pulposus along the annulus fibrosus of degenerative intervertebral disc. In addition, these granulation tissues and nerves are easily affected by interstitial changes and inflammatory mediators, thus causing pain (2). Mechanical compression of nerve roots was previously considered the main cause of low back pain. However, in actual clinical practice, for a vast majority of patients, low back pain was caused by non-nerve root compression, while DLBP was one of the main causes of non-nerve root compression (44).

Currently, the treatment methods for DLBP include non-surgical treatment (drug therapy, physical therapy), surgical treatment (intervertebral fusion, disc replacement), and minimally invasive interventional treatment (intervertebral disc injection therapy and epidural injection) (45–50). However, many of these treatments have disadvantages such as adjacent segment disorder after fusion, secondary surgery, drug dependence and tolerance, infection, dural rupture, and nerve injury. Therefore, safer PRP injection therapy, which is dedicated to repair the intervertebral disc itself, has been proposed.

**Figure 1:**
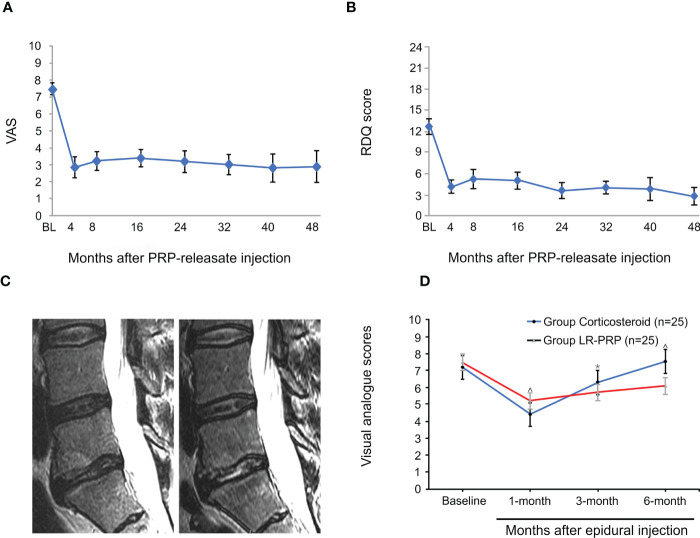
PRP for treatment of discogenic low back pain. Mean Visual Analogue Scale (VAS) score **(A)** and mean Roland-Morris Disability Questionnaire (RDQ) score **(B)** of patients after intradiscal injection of PRP. **(C)** Sagittal MRI one year before the caudal epidural and intraductal PRP injection showed increased T2 nuclear signal intensity and decreased type I Modic change in the L5-S1 disc compared to pre-injection. **(D)** Mean VAS scores were significantly lower at 3 and 6 months in patients who received LR-PRP injection. Reproduced with permission from (51–53). *P < 0.05 and ^P < 0.01.

With the development of experimental research, PRP has been used in the treatment of DLBP and has achieved good results. In a preliminary clinical trial to determine the efficacy and safety of autologous PRP releasers in patients with DLBP, Akeda et al. included 14 patients with certain inclusion criteria and evaluated the results using a visual analog scale (VAS), Roland-Morris Disability Questionnaire (RDQ), and X-ray and magnetic resonance imaging (MRI) (T2-quantification) for data analysis. During the 10-month follow-up period, the patient VAS and RDQ scores decreased significantly in the first month and continued throughout the observation period (**Figures 1A, B**). However, MRI quantitative analysis did not observe that this method had a significant effect on the repair of intervertebral disc, but it did not have a negative effect on the height of intervertebral disc. Furthermore, no adverse effects were observed during follow-up (51). In a case report, MRI examination was performed 1 year after PRP injection, and the results showed that the nuclear T2 signal intensity increased (**Figure 1C**), notably, and pain and function were also significantly improved (52). However, only one patient was described in this report, and a clear correlation between disc improvement and PRP injection could not be determined. Therefore, it is necessary to expand the sample size and carefully design future prospective studies. In order to prove the efficacy of PRP, Zhang et al. showed that intradiscal injection of PRP can significantly relieve pain and improve lumbar function in patients with DLBP (54). In another randomized, double-blind controlled trial, Ricardo et al. included 50 eligible patients with low back pain. All patients were randomized 1:1 to corticosteroids and leukocyte-rich PRP (LR-PRP) groups and were treated by tail epidural injection. Pain levels and quality of life were evaluated using the VAS and the Short Form 36-Item Health Survey (SF-36) at 1, 3, and 6 months after treatment. The results showed that the VAS scores of both groups were significantly lower than those before treatment. The corticosteroid group had lower VAS scores at 1 month, whereas the LR-PRP group had lower scores at 3 and 6 months (**Figure 1D**). SF-36 at 6 months showed a significant improvement in all domains in the LR-PRP group. Both groups showed good therapeutic effects in pain relief, but LR-PRP was superior to corticosteroids in improving the quality of life (53). Tuakli-Wosornu et al. reported that in a randomized controlled trial of 72 patients, compared to those of a control group that received contrast media alone, the functional rating index, the numeric rating scale (NRS), and the North American Spine Society satisfaction scores of the PRP group were significantly improved (55). But in a recent trial, Zielenskietal’s findings confirmed that PRP did not work as well in DLBP, with only 17% of patients showing significant improvement in clinical symptoms. This may be due to patient demographic differences, sensitivity to outcome measurements, or misalignment of statistical analysis (56).

### 3.2 PRP for treatment of lumbar disc herniation

Lumbar disc herniation (LDH) is one of the most common causes of low back pain (57). LDH is a disease caused by a combination of genetic, aging, biomechanical, and environmental factors (58). In terms of pathophysiology, LDH is usually due to IVD degeneration, which tends to develop with age. Disc lesions can develop in young adulthood. Subsequently, due to the gradual decrease in water content in the annulus fibrosus (AF) and nucleus pulposus (NP), the tension decreases, and the height of the intervertebral disc decreases, resulting in narrowing of the intervertebral space, loss of elasticity of the NP, and relaxation of the structure of the intervertebral disc. Dehydration may further aggravate cracks in AF. Subsequently, degenerated NP can highlight fissures or weaknesses in AF due to injury or trauma. Prominent NP can become ischemic and cause nerve root symptoms through mechanical compression, chemical stimulation, and activation of the inflammatory cascade (59).

Clinical symptoms often determine treatment options for LDH, including conservative and surgical treatment. However, the latter treatment only temporarily relieves pain or removes the NP of nerve root compression and prevents reherniation, which only partially solves problems associated with low back pain, and does not promote disc regeneration or restore its biomechanical properties such as damping and vibration reduction. Therefore, PRP with endogenous healing effects may be an effective treatment for LDH.

**Figure 2:**
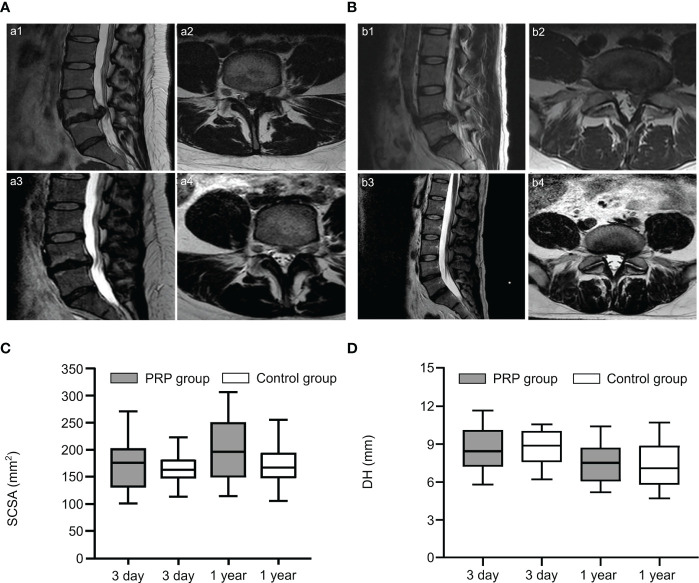
PRP treatment of lumbar disc herniation. **(A, B)** Sagittal and axial slices showed significant disc resorption before and after epidural injection of PRP in two patients. Sagittal (a1, b1) and axial (a2, b2) MRI images showed lumbar disc herniation in two patients before treatment; Sagittal (a3, b3) and axial (a4, b4) images showed significant disc resorption after treatment. **(C, D)** Comparison of spinal cord cross-sectional area (SCSA) and disc height (DH) on MRI at 3 and 1 years after treatment. Reproduced with permission from (60, 61).

This treatment has been reported in clinical practice. Benjamin described a case report of two patients with symptomatic disc herniation who showed almost complete disc absorption on MRI after two epidural injections of PRP (**Figures 2A, B**) (60). Furthermore, the clinical symptoms and function of both patients improved significantly. In another prospective, randomized, controlled trial involving 60 patients, Jiang et al. randomized patients to receive transforaminal endoscopic lumbar discectomy combined with injection of PRP and without injection of PRP. During the postoperative follow-up period, VAS for lower back and leg pain and Oswestry disability index were lower in the PRP group than in the control group. Furthermore, quantitative MRI analysis showed that in the PRP group, values were 5% higher than those in the control group after treatment (**Figure 2D**). Similarly, the spinal cross-section of the PRP group was 9% higher than that of the control group (**Figure 2C**) (61). Similar results were also confirmed by Rohan et al. (62). Through clinical trials, Xu et al. found that during the 1-year follow-up period after transforaminal injection of PRP and steroids in the treatment of LDH, VAS, pressure pain thresholds (PPTs), Oswestry disability index (ODI), and SF-36 scores improved significantly compared to those before treatment, but there were no significant differences between the two groups. This suggests that the two treatments achieved similar results, but PRP may serve as a safer alternative treatment (63). No adverse events were reported during treatment in these studies.

### 3.3 PRP for treatment of spinal cord injury

Spinal cord injury (SCI) is a devastating pathological condition of the central nervous system that causes sensory, motor, and autonomic nervous dysfunction in the limbs below the injured segment (5). This intractable disease causes unavoidable physical, psychological, and economic burden on patients and their families (64). Due to the complex pathophysiological processes after SCI, such as a series of cascades of ischemia, inflammation, oxidative stress, and apoptosis, successful cure of SCI remains a great challenge for clinical workers (65).

Currently, SCI treatment includes early drug and surgical treatment. The former includes methylprednisolone (66), gangliosides (67), calcium antagonists (68), and dehydrating agents (69). Drugs play a key role in inhibiting lipid peroxidation, maintaining ion balance, improving circulation, alleviating edema, inhibiting the release of toxic substances, promoting axon growth, and improving functional recovery of patients with SCI. However, only hormones and gangliosides have been confirmed in laboratory and clinical trials of neurotherapy and rehabilitation. Further, hormone therapy also has drawbacks and remains controversial due to many systemic adverse reactions (70). Early surgical treatment of SCI includes fracture reduction, orthopedics, spinal canal decompression or dilation, fixation, and bone graft fusion. The aims of surgical intervention include: (1) reconstruction of the stability of the spine, enabling patients to move early, which is conducive to more meticulous nursing, and reducing early complications and mortality; (2) prevention of secondary injury of the spinal cord caused by spinal instability; and (3) after decompression, spinal cord compression is relieved to create a relaxed internal environment for spinal nerve recovery (71–73). These methods have played a certain role in alleviating secondary nerve injury and reducing the mortality of patients after SCI, but their role in promoting functional recovery is limited because the nonregenerative nature of neurons renders SCI treatment more challenging. Preclinical trials on SCI repair are being continuously developed and some therapeutic results have been achieved. Examples include cell therapy (74) and neural tissue engineering (75). The treatment of SCI with PRP is also being investigated.

**Figure 3:**
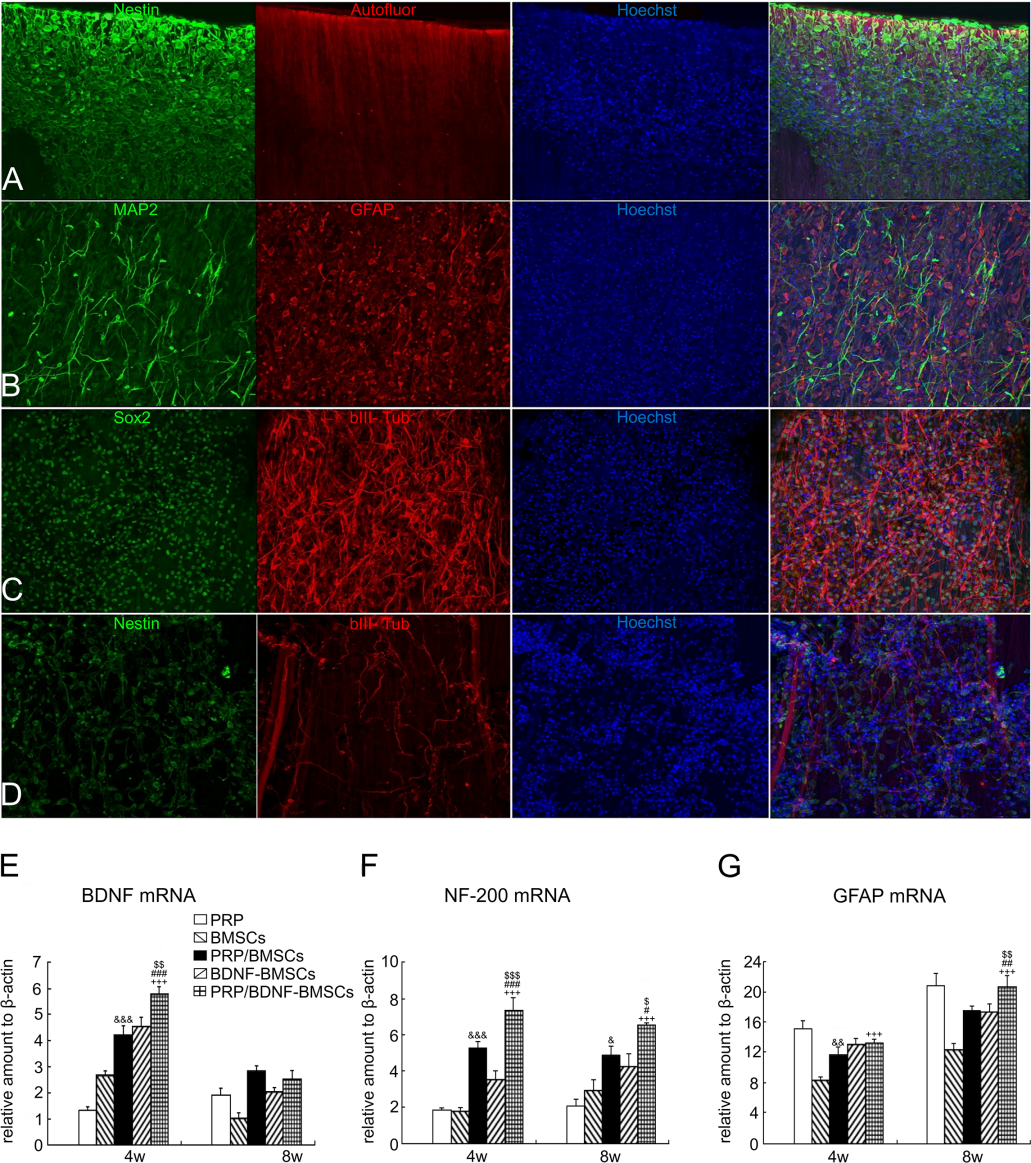
PRP treatment of spinal cord injury. **(A-C)** drNPC cultured on the two component SPRPix matrix (PRP hydrogel embedded into the rSS-PCL scaffold) demonstrated a high level of adhesion and neuronal differentiation: drNPC completely covered the entire SPRPix matrix surface and formed long βIII-tubulin- and MAP2- positive processes oriented along the spidroin microfibrils. **(D)** No directional growth was observed in scaffolds without PRP. **(E–G)** The expression of BDNF, NF-200 and GFAP genes was significantly increased after PRP and BMSCs were used together. Reproduced with permission from (77, 78). $ or $$ or $$$ P< 0.05 or P< 0.01 or P< 0.001 (PRP/BDNF-BMSCs vs. BDNF-BMSCs), # or ## or ### P< 0.05 or P< 0.01 or P< 0.001 (PRP/BDNF-BMSCs vs. PRP/BMSCs), +++ P < 0.001 (PRP/BDNF-BMSCs vs. BMSCs alone),& or && or &&&P < 0.05 or P < 0.01 or P < 0.001 (PRP/ BMSCs vs. BMSCs alone).

**Figure 4:**
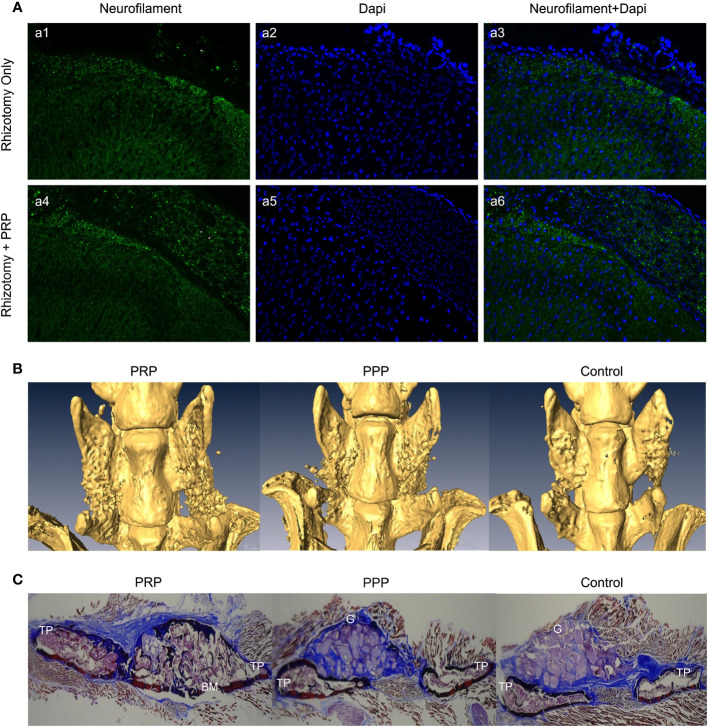
**(A)** Immunofluorescence analysis of neurofilament in the spinal cord dorsal horn and roots 8 weeks following dorsal rhizotomy and repair. (a1-a3) The Rhizotomy Only group showed obvious dorsal root degeneration. (a4-a6) Rhizotomy + PRP group showed obvious dorsal root repair. Degenerated dorsal roots are indicated by (*), and repaired dorsal roots are indicated by (**). **(B)** Microscopic CT scans of each group. PRP group had better fusion quality between transverse processes. **(C)** Masson’s trichrome staining showed more new bone formation in the PRP group with mature fusion masses between transverse processes. TP transverse process, BM bone marrow, G granules of mineral carrier. Reproduced with permission from (79, 80).

In an *in vitro* trial, Michiko et al. co-cultured human PRP with the cortex and spinal cord of 3-day-old Sprague-Dawley rats and added neutralizing antibodies against different growth factors. They found that PRP promotes spinal cord axon growth through mechanisms related to IGF-1 and VEGF, while inhibition of TGF-β1 had a negative effect on axon growth (76). In another trial, PRP was combined with BDNF-overexpressing BMSCs in a rat model of spinal cord hemisection. In the treatment group, the neuronal gene markers NF-200, GFAP, and MAP2 were highly expressed in the presence of PRP, thus promoting axonal myelin sheath regeneration (**Figures 3E–G**) (77). Baklaushev et al. designed a novel two-component matrix (SPRPix) that contained spider silk proteins and PRP. This complex promoted the proliferation of directly reprogrammed human neural precursor cells (drNPC) in neurons, astrocytes, and other neural support cells, and allowed these β III-tubulin- and MAP2-positive nerve cells to grow oriented along the spider protein microfibers (**Figures 3A–C**). In contrast, there was almost no directional growth in the hydrogels when PRP was removed (**Figure 3D**). Quantitative analysis of cells using the NIS Elements software (Nikon) showed that the absolute number of neuronal progenitor cells in the PRP group was five times higher than that of the blank group in the same volume. In *in vivo* experiments, drNPCs and the complex were implanted in the brain and spinal cord of rhesus monkeys, which showed good biocompatibility and successful differentiation into MAP2 positive neurons (78). Injury to the dorsal spinal cord can lead to a loss of transmission of proprioceptive information and affect motor behavior. Baklaushev et al. conducted research on dorsal spinal cord repair. The research team injected human PRP into the unilateral spinal cord dorsal root injury model and evaluated the recovery of reflex arc by electronic von Frey weekly. The arc recovered and the paw withdrawal reflex was partially restored as the treatment progressed. Intermediate filaments (nerve filaments) were observed throughout the reimplantation area (**Figure 4A**), indicating nerve regeneration. Furthermore, this treatment has immunomodulatory properties that can effectively improve regeneration and reduce inflammatory responses (79). In another study, researchers injected PRP directly into the injured spinal cord of rats and examined the effects of PRP on spinal cord repair. The results showed that PRP could protect white matter, improve motor recovery, promote neuronal regeneration and angiogenesis, and regulate its size (81). These experiments indicated that PRP could be used as a promising therapeutic agent for SCI.

### 3.4 Treatment of spinal fusion

As a mature surgical technique in spinal surgery, spinal fusion aims at achieving mid- and long-term stability of the spine, which is crucial for maintaining normal spinal function. This can be achieved using different surgical methods, implants, and grafts (82). Spinal fusion has been widely used in the treatment of various spinal malformations and diseases, including spondylolisthesis, fractures, lateral curvature, and lumbar disc diseases (83–88).

However, surgical complications, especially failure of postoperative bone formation, connection, or reshaping, results in the formation of a nonunion or pseudoarthrosis. This condition is significantly more frequent in multisegmental and osteoporotic cases (89). Currently, various materials have been used in clinical practice to improve the rate of bone fusion. The most used products include autologous, allograft, and bone graft replacement materials. Autografts have long been considered the gold standard bone graft material and have significantly improved bone fusion rate (90). However, autologous bone grafting presents associated safety issues such as donor site-related complications (especially at the iliac crest), limited bone harvesting, increased blood loss, and increased operative time (91). In other studies, allografts have proven to be promising materials that can promote bone formation. However, allografts have also been questioned as therapeutic options and are considered less successful than autografts (92). In recent years, the application of various alternative bone grafting materials combined with mesenchymal stem cells and osteogenic induction factors, such as bone transplantation, has a bright prospect. These biomaterials exhibit good biocompatibility and are beneficial for cell attachment, migration, and osteogenic matrix deposition. Good porosity is suitable for the development of the vascular network and the diffusion of nutrients (93). PRP, a natural growth factor library, has also been used for spinal fusion.

**Figure 5:**
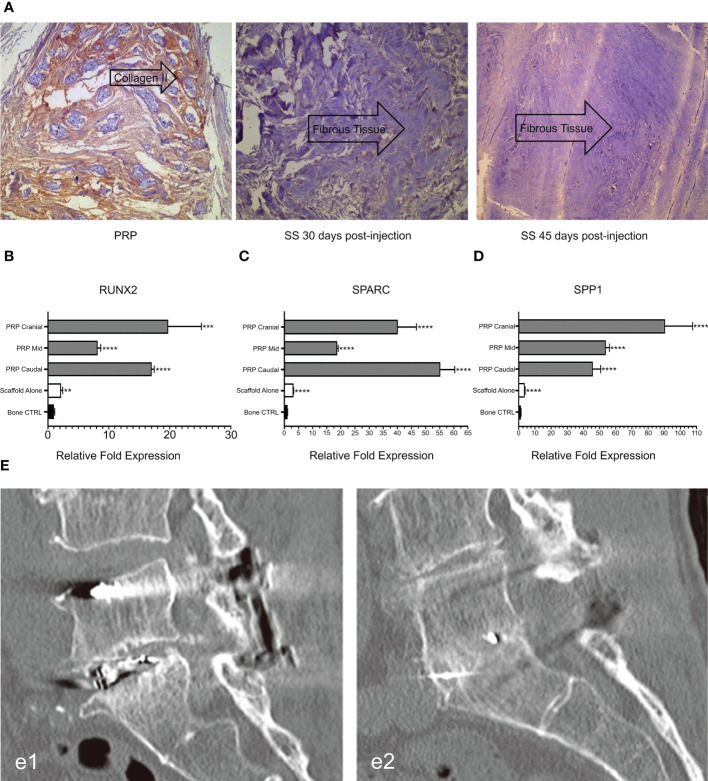
**(A)** Immunohistochemical expression of type II collagen in intervertebral disc tissues at different time points after injection. **(B-D)** Analysis of osteogenic gene expression. **(E)** CT evaluation of spinal fusion in different groups. Reproduced with permission from (96–98). **P = 0.0034, ***p = 0.0002, ****p < 0.0001. e1: Control group. e2: PRP group.

Related studies have shown that TGF- β and PDGF contained in PRP can attract osteoblast progenitor cells to the desired site for value-added differentiation and promote bone formation. Simultaneously, VEGF in PRP can induce angiogenesis and provide sufficient nutrients for the osteogenic process to promote bone fusion (94, 95). The application of PRP, alone or in combination with composite materials, has been reported in animal models and clinical trials. In a rat lumbar posterolateral fusion model, PRP derived from rat peripheral blood was combined with collagen-mineral scaffolds for treatment. After 12 weeks, micro-computed tomography (CT) and manual palpation showed a more efficient fusion rate than the control group (**Figure 4B**), and Masson’s trichrome staining showed mature bone fusion blocks and more new bone formation between the transverse processes in the PRP group (**Figure 4C**) (80). However, no further studies have been conducted on the mechanisms promoting integration. This has been explained in a previous study. Jeffrey et al. constructed composite scaffolds based on a combination of hydroxyapatite/type I collagen (MHA/Coll) and PRP. *In vivo*, the composite group produced more trabecular and cortical bone than did the stent group alone, and the fusion rate was better than that of the control group. Quantitative analysis of the expression of key osteogenic genes showed that RUNX2, SPARC, and SPP1 were significantly up-regulated compared to the control group (**Figures 5B–D**) (96). This appears to be the key to the promotion of bone fusion. With the advent of the aging society, the spinal fusion rate of elderly patients with degenerative spinal diseases due to osteoporosis is not satisfactory (99). To this end, researchers have combined collagen-binding bone morphogenetic protein-2 (CBD-BMP-2), which has good biological activity and slow-release function, with PRP to treat osteopenia in an elderly rat model. Through comparative analysis, the fusion rate, area, volume, and number of bone trabeculae were significantly better than those of the control group; however, PRP alone did not show satisfactory fusion efficiency (100). This finding is similar to that of a meta-analysis of clinical cases (101). The study concluded that additional use of PRP did not lead to further significant improvement in patients compared to traditional graft techniques and that PRP may have a limited role in enhancing spinal fusion. However, several other retrospective and meta-analyses have confirmed the role of PRP in promoting new bone formation and bone fusion (102, 103). In a prospective randomized controlled trial to evaluate PRP efficacy after posterolateral lumbar fusion surgery, Kubota et al. randomly divided 62 patients into PRP and control groups; the final bone union rate, fusion area, and mean union time in the PRP group were significantly better than those of the control group at the end of follow-up period (104). In another retrospective analysis, 20 patients underwent single-level transforaminal lumbar interbody fusion (TLIF) surgery for L4 spondylolisthesis, among which, 11 patients received PRP combination therapy. Postoperative CT analysis showed that the bone fusion effect was significantly better in the PRP group than in the control group (**Figure 5E**); however, there was no significant difference in healing time (97). The differences in the above experiments may be related to the preparation method, composition, concentration, and injection dosage of PRP, which should be further studied.

### 3.5 Promotion of degenerative disc regeneration

IVD degeneration (IVDD) is a serious global health concern. In 2019 alone, the global prevalence rate exceeded 60%, with a higher prevalence among men and the elderly, resulting in high healthcare costs for families and societies (4). The pathophysiological process of IVDD involves dehydration of the NP, tearing of the AF, rupture of the cartilage endplate, and inflammatory factors (105). These cascade reactions are often the root of discogenic pain and LDH, which can affect the extension and flexion of the spine and, in severe cases, lead to disability or even loss of work force (106). Due to the avascular tissue structure of the IVD, its self-healing ability is extremely poor and common drug therapy is generally ineffective. Currently, IVDD treatment methods include mainly bed rest, epidural injection, physical therapy, surgical decompression, disc fusion, and disc replacement (107).

However, clinical treatment (including conservative and surgical methods) is still aimed at alleviating symptoms rather than limiting disease progression or preventing disease according to etiology. Therefore, it is necessary to develop new treatment strategies. In recent years, regenerative biotherapy targeting IVD regeneration, including gene therapy, stem cell injection, and growth factor tissue engineering, has become the focus of recent research (108). PRP is also being investigated for this purpose. Related studies have shown that growth factors (TGF- β 1, VEGF, PDGF, and EGF) and adhesive proteins (fibrin, fibronectin, and vitronectin) in PRP can stimulate NP cell proliferation and extracellular matrix regeneration. This is helpful for intervertebral disc repair and symptom relief in early IDD patients (4).

**Figure 6:**
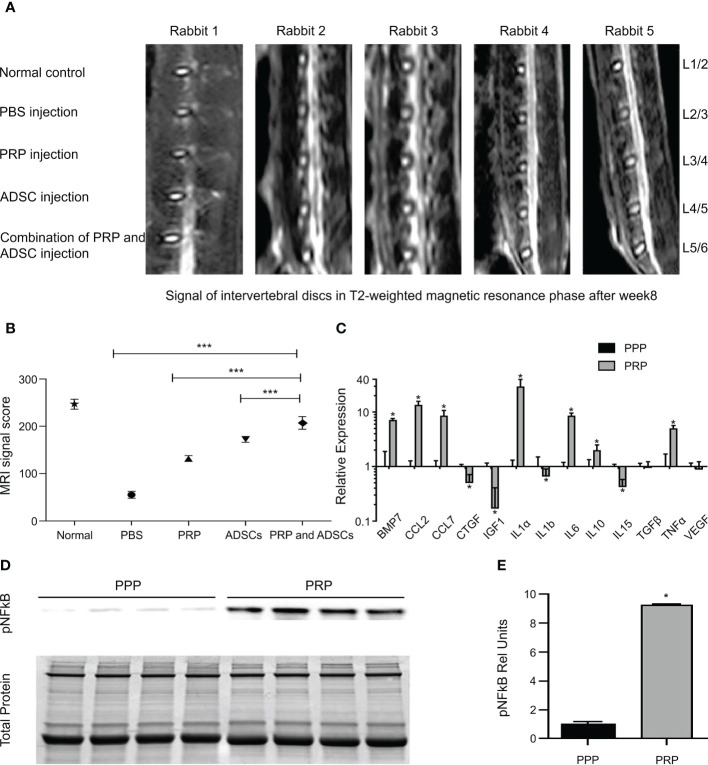
**(A)** The disc signals in magnetic resonance T2-weighted image in the group of ADSC-combined PRP showed higher signal strength than the other groups. **(B)** PRP combination with ADSCs has the best effect in reversing degeneration. **(C)** Gene expression of growth factor and cytokine transcripts from PPP or PRP treated tendon fibroblasts. **(D)** Immunoblots for phospho NFκB activation from tendon fibroblasts treated with PRP or PPP. **(E)** Band densitometry analysis for phospho NFκB blots. Reproduced with permission from (111, 112). * P< 0.05, *** P<0.001.

Gelalis et al. injected autologous PRP into a rabbit IVDD model and found that the degree of degeneration of AF and NP was significantly lower in the PRP group than in the control group during the 6-week follow-up period. Notably, the histological analysis of hematoxylin-eosin staining in the treated group showed a significant increase in type II collagen expression (**Figure 5A**), which resulted in significant reversal and regeneration of the affected disc (98). In the artificial simulation of NP, alginate, a highly customizable biocompatible polymer whose properties can be adjusted, is used. Growney et al. combined PRP with alginate chemistry using carbondiimine chemistry, and the obtained a hydrogel that had good biocompatibility and mechanical properties. After 14 days of co-culture with human nucleus pulposus cells (hNPC), quantitative analysis of glycoaminoglycan showed that the complex allowed ECM expression, suggesting that PRP-modified alginate could stimulate matrix synthesis and repair (109). PRP also restored glycosaminoglycan levels in another study (110). In a cell therapy trial, the researchers combined PRP with adipose tissue-derived stromal cells (ADSC) to treat a New Zealand rabbit model of early disc degeneration. After 4 weeks, the signal strength and disc height on T2-weighted MRI were assessed. The disc signal in the ADSC combined with PRP group showed higher intensity than that in the other groups (**Figure 6A**) and was statistically significant in reversing disc degeneration (**Figure 6B**) (111). Similar therapies based on the combination of PRP and stem cells have been reported in many studies (113–116), and the biological efficacy of PRP has been confirmed in the experimental results. With the addition of PRP, many types of stem cells can differentiate into NP cell phenotype, and the diseased intervertebral disc can be reversed and regenerated to a certain extent, which provides a potential feasible scheme for future clinical treatment.

### 3.6 Promotion of ligament injury repair

The spine ligaments carry most of the tension load on the spine and their carrying capacity is the strongest when the load direction is in line with the direction of the fibers. When the spinal segment is in motion, the corresponding ligaments are stretched and stabilize the spine (6). The spinal ligaments have many functions. First, spinal ligaments provide sufficient physical activity between the two vertebral bodies to maintain a certain posture. Second, they protect the spinal cord by limiting movement, maintaining it within the proper physiological range, and absorbing energy. Third, by limiting displacement, they absorb high load and highspeed energy to protect the spinal cord from injury (117, 118). Therefore, the treatment of ligament injuries plays a vital role in maintaining spinal stability. Autologous PRP was administered to a rabbit model mimicking spinal ligament injury and compared with a saline-injected control group. Better healing was observed in the morphology and histology of the treated ligaments, which was determined to be achieved through activation of inflammatory pathways (IL-17 and TNF) (119), by pathway analysis. This idea was partly supported by another animal study. Hudgens et al. analyzed the effect of PRP on gene expression changes in tendon fibroblasts by microarray and analyzed multiple changes in gene expression using the Ingenuity Pathway Analysis software. They concluded that PRP induced a large number of inflammatory responses and oxidative stress in fibroblasts through activation of the TNF-α and NFκB signaling pathways, leading to tissue regeneration responses (**Figures 6C–E**) (112). The supraspinal ligament (SSL) and interspinous ligament (ISL), as part of the posterior ligament complex, play an indispensable role in spinal stability. A case report described two cases of SSL and ISL injury, in which, ultrasound-guided ligament-targeted injection of LR-PRP leukocyte-rich plasma was successful, with almost 100% pain improvement 6 and 9 months after treatment, respectively (120).

### 3.7 PRP for treatment of sacroiliac joint disease

Another common cause of lower back pain is sacroiliac joint (SIJ) disease, which is considered the most likely source of lower back pain in patients after lumbosacral or lumbar fusion surgery (121). Initial treatment of SIJ disease is usually conservative and includes physical therapy, massage therapy, and drug therapy. However, these treatments are often aimed at pain relief, rather than eradication. Other treatment options, such as periarticular injection, intraarticular injection, or nerve block, are generally administered if there is no improvement in symptoms after 6 weeks of conservative treatment. Surgery is considered when all treatments fail (7). PRP can be used as a new treatment modality for the treatment of SIJ disease.

This concept has been tested in several clinical trials. In a prospective randomized trial, Singla et al. found that PRP efficacy was maintained over a 3-month follow-up period, with VAS scores reduced to 90%, while efficacy in the steroid group was reduced to only 25% over the same time period. The results of Modified Oswestry Disability Questionnaire (MODQ) and SF-12 also confirmed the efficacy of PRP (122). Patrick et al. reported similar results (123). This may be due to the abundance of growth factors in PRP, which enhances the biological environment and contributes to tissue homeostasis. Notably, in one case report, the efficacy of PRP maintained improvement in joint stability and low back pain after 4 years of treatment (124). Therefore, according to clinical trials, it can be concluded that ultrasound-guided PRP injection is a safe and effective treatment for SIJ disease and can reduce dysfunction and low back pain.

### 3.8 PRP for treatment of related complications

Related complications of spinal disease also plague healthcare professionals and pose a significant risk to patient outcomes, including shoulder pain, deep surgical site infections, pressure ulcers, and epidural fibrosis. Therefore, treatment of complications is of great importance for the good prognosis of patients with spinal diseases.

Shoulder pain is one of the most common complaints in patients with spinal cord injuries in wheelchairs. The common causes include shoulder tendinopathy and other musculoskeletal disorders. In a prospective trial of six patients with chronic shoulder pain due to rotator cuff disease, Dyson-Hudson et al. treated the lesion with ultrasound-guided injection of PRP. During the 24-week follow-up period, the wheelchair user shoulder pain index, pain NRS, and the physical examination scores showed a downward trend. The overall condition of the patient improved and no adverse events were reported (125). The efficacy of PRP has also been demonstrated in shoulder pain caused by biceps tendinopathy (126).

Deep surgical site infections (dSSI) undoubtedly pose significant therapeutic challenges for spinal surgeons, with post-infection treatment typically focused on surgical debridement, wound drainage, and long-term antibiotic therapy. Vasilikos added PRF to 12 patients at the time of the second surgical revision. All patients achieved complete wound healing between 14 and 21 days, and no recurrence of dSSI or complications were observed during follow-up (127).

**Figure 7:**
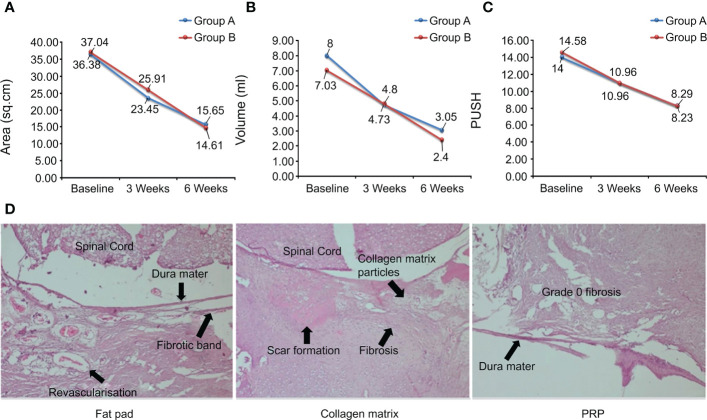
**(A-C)** Ulcer area, ulcer volume, and PUSH score over time. **(D)** Scar and fibrotic tissue staining in different groups. Reproduced with permission from (130, 131).

Pressure ulcers (PrU) is one of the main complications of SCI. PrU may be more difficult to heal in patients with SCI because such patients often require intensive care around the clock. This is certainly a daunting challenge for patients and caregivers. PrU often does not heal and leads to progressive chronic inflammation, which is the root cause of death in SCI patients (128). PrU healing is a dynamic and complex process, and many growth factors in platelets can regulate this complex event and improve the quality of life and prognosis of patients. In a pilot study of 15 patients with SCI and chronic PrU who developed fistulas, the authors observed a decrease in fistula secretion levels after 1 week of PRP treatment, no fistulas at 2 weeks, and complete disappearance of fistulas at 3 weeks on MRI (129). Gurpreet et al. demonstrated in comparative tests that PRP significantly improved ulcer area, volume, and pressure ulcer healing scale scores compared to hydrogel dressings (**Figures 7A–C**), and had significant advantages in granulation, epithelial formation, and vascular formation (130). Similar experiments have also demonstrated the feasibility of PRP in the treatment of PrU (128, 132, 133). PRP may be a better alternative to traditional dressings for the treatment of PrU.

Lower back pain after laminectomy is a recurrent clinical condition that has attracted the attention of spinal surgeons. This is caused by epidural fibrosis, which occurs naturally after laminectomy. Excessive scarring produces adhesion between tissues or stress on surrounding anatomical structures, leading to clinically significant sequelae. Therefore, effective prevention of epidural fibrosis is conducive to patient prognosis. In an animal study, Guler et al. performed laminectomy in male rats and covered the exposed dura with fat pads, collagenous dura matrix, and PRP. At the end of the week 4, the three groups of rats were sacrificed and the samples were histologically analyzed. Epidural fibrosis (grade 3, 43%) was more common and presented the largest area of fibrotic scar tissue in the collagen-dural matrix group, while no fibroblast activity was detected in PRP group (grade 1, 71%; grade 0, grade 2, 14%). The PRP group prevented epidural fibrosis more efficiently than the fat pad group (grade 1, 71%; grade 2, 28%) and also showed better results (**Figure 7D**) (131). Another study investigated the barrier functions of hyaluronic acid (HAS), activated polyethylene glycol, polyethylene imine (PEG), and PRP. Histopathological results showed that PRP could improve tissue integrity and reduce scar tissue level, histopathological grade, Masson’s trichrome staining (MTS) grade, and fibroblast numbers. Real-time polymerase chain reaction showed that PRP reduced the levels of type I collagen, type III collagen, TGF-1β, and TNF-α compared to the control group. These results indicated that PRP could significantly reduce the level of peridural fibrosis in a rat model (134).

## 4 Safety study of PRP therapy

The safety of biological agents is very important for patients, and only when safety is guaranteed can better efficacy be achieved. In a systematic review of PRP for the treatment of skin aging, PRP accelerated skin healing and reduced signs of skin aging after laser surgery with no reported serious adverse events (135). In the field of dermatology, Shen et al. applied PRP to the treatment of skin ulcers and concluded that PRP was superior to traditional treatments owing to its high healing rate, high percentage of area loss, and smaller vascular ulcers. However, while PRP showed fewer adverse events in the short term, it showed a higher rate of adverse events in the long term (136). In a study by Nassar et al., the PRP group showed greater side effects and no significant improvement in the treatment of atrophic scars compared with patients receiving carboxyl therapy (137). In burn departments, PRP has played an important role in the repair of severe burn wounds and its safety has also been affirmed (138). In the orthopedic field, Taisuke et al. applied PRP for the treatment of ankle osteoarthritis. During the 24 weeks of treatment and follow-up, PRP did not cause any serious adverse reactions and significantly reduced pain, demonstrating the safety and efficacy of the treatment with PRP (139). PRP has also been shown to be safe and effective in similar studies on knee osteoarthritis (140).

Based on the above studies, overall PRP presents good efficacy in different fields, and most studies have shown that its safety can be guaranteed. However, some studies have also found side effects that cannot be ignored. Few in-depth studies and reports on the safety of PRP in spine surgery have been published. If PRP is to be used in spinal surgery, a large number of studies are needed to confirm its safety.

## 5 Conclusions and prospects

As an indispensable part of the skeletal system controlling body movements, the spine plays an important role in uploading and distribution. On being affected by a serious disease, it results in immeasurable serious consequences. Spine-related diseases are of great concern owing to their increasing annual incidence. Although the medical community has developed a variety of treatment methods, including surgical, conservative, and interventional treatment, there is no specific treatment for common diseases in spinal surgery. At present, several problems need to be addressed. For example, (1) bone nonunion and pseudarthrosis after spinal fusion, (2) accelerated degeneration of intervertebral disc, (3) how to promote intervertebral disc regeneration and restore its biomechanical properties after minimally invasive surgery, (4) how to promote nerve growth and reconnection after spinal cord injury, (5) how to avoid recurrent attacks of low back pain, (6) how to treat various complications caused by spinal column-related diseases and improve patients’ prognosis and survival rate. All these are the key problems that need to be solved in this field.

This article reviewed the applications of PRP in the treatment of spinal diseases. PRP is an autologous product with platelet concentrations much higher than baseline. The therapeutic principle of PRP is that injection of PRP at the site of injury or surgery can initiate tissue repair by releasing a variety of bioactive factors (PDGF, TGF-β, VEGF, and IGF-1) and adhesive proteins in α particles, promoting revascularization and the generation of new connective tissue. This accelerates the healing of chronic injuries and repair of acute injuries. This process is attributed to the synergistic activity of various growth factors, cytokines, and local regulators *via* paracrine, endocrine, and autocrine mechanisms. PRP preparations are becoming increasingly popular due to their widespread use in different medical fields. The main advantages of PRP include its safety and the superior technology of commercial equipment for the preparation of PRP biologics that can be widely used. Most importantly, PRP is an autologous product with no known side effects and shows promising therapeutic efficacy in a wide range of medical fields.

However, there is still considerable work to be done to achieve widespread application in the field of spine-related diseases. For example, (1) standard procedures for PRP production are lacking. Currently, there are many formulations and techniques to produce PRP, but there is no standard for these parameters, including initial whole blood volume, platelet concentration, and PRP composition. (2) Individual differences. For example, age differences can lead to differences in growth factors and the overall composition of PRP. Osteoporosis can lead to differences in treatment effects. (3) Insufficient number of experiments. In both clinical trials and animal experiments, there is a low level of evidence, insufficient sample size, confusion in nomenclature, lack of standardization of preparation methods, and lack of in-depth research on basic science. (4) There is no clear consensus on indications, and (5) the cost of treatment remains controversial. Some researchers believe that short-term use of PRP is more expensive than steroid injection, while long-term treatment may be economical. Finally, the (6) lack of safety studies. Moreover, there is no uniform standard for safety assessment. Faced with these issues, spinal surgeons should conduct more clinical, randomized, controlled, and unbiased trials and a large number of animal trials with more in-depth investigation to reach a consensus on the standardized classification of PRP. Formulation of a more systematic and authoritative production and treatment standards is required. The treatment of spine-related diseases still in its early stages; however, with further understanding of PRP and the advancement of manufacturing technology, it is possible to achieve a standardized treatment plan for spine surgery in the future.

## Author contributions

HW wrote the initial manuscript. CF and YX contributed new ideas. HW and YL created the figures. YX and JZ created **Table 1** HW, JZ, YX, and YL revised the manuscript. All authors contributed to the article and approved the submitted version.
